# Ultrasound‐Based Knee Osteoarthritis Severity Assessment and Its Association With Kellgren–Lawrence Grading

**DOI:** 10.1155/tswj/2248441

**Published:** 2026-01-24

**Authors:** Rita Vivera Pane, Handayani, Aisyah, Nurhasyimah Hisamud-Din, Aufar Zimamuz Zaman Al Hajiri, Alif Noeriyanto Rahman

**Affiliations:** ^1^ Department of Physical Medicine and Rehabilitation, Faculty of Medicine, Universitas Nahdlatul Ulama Surabaya, Surabaya, East Java, Indonesia; ^2^ Department of Physical Medicine and Rehabilitation, Haji General Hospital of East Java Province, Surabaya, East Java, Indonesia; ^3^ Faculty of Medicine, Universitas Nahdlatul Ulama Surabaya, Surabaya, East Java, Indonesia; ^4^ Department of Rehabilitation Medicine, Hospital Tuanku Ja′afar, Seremban, Negeri Sembilan, Malaysia, moh.gov.my; ^5^ Musculoskeletal Pain Intervention and Regeneration, Faculty of Medicine, Universitas Padjadjaran, Sumedang, West Java, Indonesia, unpad.ac.id

**Keywords:** cartilage damage, conventional radiography, Kellgren–Lawrence grading, knee osteoarthritis, knee osteoarthritis ultrasound grading

## Abstract

**Background:**

Kellgren–Lawrence (KL) grading is a conventional radiography (CR)‐based system commonly used to assess osteoarthritis (OA). Knee OA is characterized by damage to the femoral cartilage (FC); however, CR cannot directly visualize cartilage integrity. Ultrasound (US) may be superior in detecting FC damage. This study is aimed at evaluating the US‐based knee OA severity assessment and analyze its association with KL grading.

**Methods:**

This was an analytical observational study with a cross‐sectional design which included knee OA patients. All participants underwent knee US scanning by two independent raters. The severity of knee OA was assessed based on three parameters: the contour of the femoral cartilage, the presence of osteophytes, and meniscus protrusion. The results were accumulated and interpreted into grades from 0 to 4. Association between US‐based knee OA assessment and KL grading was analyzed statistically.

**Results:**

A total of 112 knee OA patients were included in this study. The average age of participants was 61.4 years, with a higher female prevalence. Grade 3 was the most frequently recorded in both US and KL grades. The US interpretation from both raters showed a perfect agreement based on weighted kappa analysis (*κ* = 1.00). A chi‐square test showed a significant association between the US and KL grade (*p* < 0.001), with a very strong association based on the Cramér′s *V* test of 0.742 (95% CI: 0.599–0.824).

**Conclusion:**

This study demonstrates the US‐based knee OA severity assessment and was associated with KL grade.

## 1. Introduction

Conventional radiography (CR) remains a valuable diagnostic tool for knee osteoarthritis (OA). Key features assessed in CR include joint space narrowing, subchondral sclerosis, osteophyte formation, joint deformity, and subchondral cysts, which help determine disease severity and guide physicians in treatment decisions. In 1957, Kellgren and Lawrence introduced the first systematic radiographic categorization scheme for OA, which remains a widely used grading system [1, 2]. While joint space narrowing observed in CR can be used to evaluate disease severity, knee OA also involves biochemical and structural changes affecting bones, cartilage, ligaments, and muscles [3]. However, CR has limitations as a predictor of knee OA severity due to its inability to directly assess structural damage. Important pathological changes, such as meniscus extrusion, femoral cartilage (FC) loss, and other soft tissue abnormalities, are difficult to evaluate using CR [4].

Magnetic resonance imaging (MRI) is the gold standard for identifying knee OA due to its superior ability to visualize soft tissues [5, 6]. However, in developing countries, MRI availability is often limited due to high costs and restricted access, as it is typically only available in specialized healthcare facilities and referral centers. In contrast, ultrasound (US) has demonstrated excellent concordance with MRI findings in knee OA cases [7]. A study by Kauppinen et al. found a high level of agreement between MRI and US in assessing FC thickness [8]. Their research explored the potential relationship between knee OA symptoms and US findings, demonstrating that US is an accurate method for detecting morphological changes, including alterations in the medial femoral condyle cartilage, knee osteophytes, and medial meniscal protrusion. However, definitive diagnostic criteria have yet to be fully established [9]. Musculoskeletal US provides a direct view of the meniscus, bursa, synovial membrane, hyaline articular cartilage, and bone cortex, making it a valuable imaging modality for evaluating various joint disorders. Additionally, US is a fast, nonradiating, and noninvasive scanning method, making it a useful tool for the early identification and monitoring of knee OA [10–12].

Currently, only a small number of doctors have begun using US devices to support their clinical practice. However, US is a valuable diagnostic tool, particularly in the musculoskeletal field, as it can be used to diagnose various diseases. Given the clinical relevance of knee OA, US is especially appropriate for assessing cartilage both quantitatively and qualitatively. Previous studies have shown that US‐based knee OA severity assessment is a reliable tool comparable to Kellgren–Lawrence (KL) grading. One study evaluating US findings alongside KL grading found that both grading systems were acceptable. Notably, the proposed US grading system demonstrated strong agreement with KL grading, and its simplicity proved to be an advantage [13]. Despite its many benefits, US grading does not provide a comprehensive assessment of knee OA. In this study, the authors propose a US‐based severity assessment for knee OA. Therefore, the aim of this study is to evaluate knee OA severity using US and analyze its association with KL grading. By implementing a US‐based severity assessment, physicians may be able to make more informed management decisions in the future, benefiting from a grading system that is simpler, more cost‐effective, and potentially more accurate.

## 2. Materials and Methods

This was an observational analytic study with a cross‐sectional design. Patients diagnosed with knee OA who were registered at the Department of Physical Medicine and Rehabilitation (PM&R), Haji Regional General Hospital of East Java Province (RSUD Haji Provinsi Jawa Timur), Surabaya, Indonesia, between October 2023 and May 2024, were recruited as study participants. Patients who met the inclusion criteria were assigned a sample registration number. Simple random sampling was used for participant selection, with randomization conducted through a website‐based application.

### 2.1. Inclusion and Exclusion Criteria

The inclusion criteria consisted of patients with knee OA who had been clinically diagnosed according to the American College of Rheumatology (ACR) criteria. Patients presenting with knee pain were included if they met at least three of the following criteria: age over 50 years, morning stiffness lasting less than 30 min, crepitus on knee motion, bony tenderness, bony enlargement, and no palpable warmth. Participants were assessed for knee OA severity using both radiographic evaluation with KL grading and musculoskeletal US scanning. The exclusion criteria included participants with a history of knee injury, prior knee surgery, or autoimmune diseases. Additionally, those who had received intra‐articular injections within the last 6 months were also excluded from the study.

### 2.2. KL grading

KL grading system is widely used to determine the severity of knee OA. The original KL classification was developed based on anteroposterior (AP) knee radiographs. The grading system ranges from 0 to 4, with higher grades indicating increasing severity of OA. The KL grades are defined as follows: Grade 0, no feature of OA; Grade 1, doubtful joint space narrowing and possible osteophyte lipping; Grade 2, obvious osteophytes and possible joint space narrowing; Grade 3, multiple moderate osteophytes, obvious joint space narrowing, some sclerosis and possible bone end deformity; Grade 4, large osteophytes, obvious joint space narrowing, severe sclerosis, and obvious bone end deformity [2, 14]. In this study, the KL grade was assessed for each participant based on their knee CR by a radiologist′s interpretation with 15 years of experience.

### 2.3. US‐Based Knee OA Severity Assessment

In this study, we proposed a US‐based knee OA severity assessment. US scans were assessed at three sites: the FC, the medial knee joint (MKJ), the lateral knee joint (LKJ), and the meniscus. Any change in FC was indicated as OA. In addition, for severity consider the presence of osteophytes in MKJ, LKJ, and the presence of meniscus protrusion. The assessment included evaluating the regularity of the FC, whereas MKJ and LKJ were assessed for the presence of osteophytes and meniscus protrusion. Osteophytes develop as a compensatory mechanism to strengthen and stabilize joints, providing protection for the articular cartilage [15]. They were detected within the scanning field, including the medial tibial condyle, lateral tibial condyle, medial femoral condyle, lateral femoral condyle, and patellofemoral joint. The meniscus is a fibrocartilaginous structure composed of circular fibers. When the knee bears a load, part of the force is directed radially. The meniscus resists this load through circular tension. If the cartilage′s shock‐absorbing function diminishes or fails, circular tension is disrupted, leading to altered load distribution and increased force. Repetitive stress can cause meniscus protrusion, characterized by a change in shape and/or position into the surrounding joint space, allowing for pressure relief as compensation [16]. Measurement of the protruded meniscus was performed by scanning at a 30‐degree flexion position.

**Figure 1:**
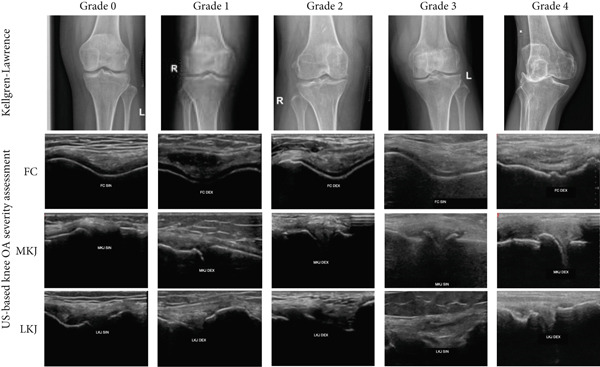
Kellgren–Lawrence grade and US‐based knee OA severity assessment illustration from Grade 0 to Grade 4 among participants. FC, femoral cartilage; MKJ, medial knee joint; and LKJ, lateral knee joint.

This grading system consists of five grades, ranging from Grade 0 to Grade 4. Grades 1–3 are determined by the presence of osteophytes: Grade 1 if osteophytes are very small or doubtful, Grade 2 if osteophytes are present and clearly seen, without meniscal protrusion, Grade 3 if osteophytes are present and clearly seen, with meniscus protrusion. Furthermore, Grade 4, absence or almost absence of FC associated with prominent osteophytes and meniscus protrusion. A more detailed description is provided in Table 1. A higher grade indicates more severe knee OA progression, similar to the KL grading system. The US grading system is illustrated in Figure 1.

**Table 1:** US‐based knee OA severity assessment description.

**Grade**	**Description**
0	The femoral cartilage is smooth and no osteophytes in the medial and lateral knee joint.
1	The irregularity of the femoral cartilage surface with minimal or doubtful osteophytes in the medial and/or lateral knee joint.
2	The irregularity of the femoral cartilage surface with prominent osteophytes in the medial and/or lateral knee joint.
3	The irregularity of the femoral cartilage surface with prominent osteophytes in the medial and/or lateral knee joint, accompanied by a protruded meniscus along part or the entire medial and/or lateral knee joint.
4	The completely loss of femoral cartilage surface in some areas with prominent osteophytes in the medial and/or lateral knee joint, accompanied by a protruded meniscus along part or the entire medial and/or lateral knee joint.

### 2.4. US scanning

US scans were performed using the Wisonic Clover 60, a 2D musculoskeletal US device with a 20 MHz linear probe (Shenzhen Wisonic Medical Technology Co. Ltd., Shenzhen, Guangdong, China). US evaluations were conducted by two raters with 6 years of experience, both certified as interventional pain sonologists (CIPS). The FC was assessed with the knee in full flexion, whereas the medial and LKJs were scanned with the knee flexed at 30°. This is the recommended neutral position for knee joint examination using US. According to several studies, the 30‐degree flexion position is the most sensitive for detecting knee pathologies, including meniscus protrusion and joint narrowing or osteophytes. [17, 18].

### 2.5. Data Analysis

Demographic data, including age, sex, and the affected knee side in OA were collected. Descriptive analysis including sensitivity: *True Positive/*(True Positive + False Negative), specificity: *True Negative/*(True Negative + False Positive), positive predictive value (PPV): *True Positive/*(True Positive + False Positive), negative predictive value (NPV): True Negative/(True Negative + False Negative), and accuracy: (True Positive + True Negative)*/*(True Positive + True Negative + False Positive + False Negative).

Inter‐rater agreement on US scan results was analyzed using a weighted kappa. The association between the US‐based knee OA severity assessment and KL grade was analyzed using a chi‐square test, with significance set at *p* < 0.05. To measure the strength of the association between US‐based knee OA severity assessment and KL grade, Cramér′s *V* test was used. Data collection was performed using Microsoft Excel 365 (Microsoft Corp., Redmond, Washington, United States), whereas statistical analyses were conducted using IBM SPSS Statistics 26.0 (IBM Corp., Armonk, New York, United States).

## 3. Results

### 3.1. Participant Data

This study included 112 participants diagnosed with knee OA. The participants had a mean age of 61.4 ± 10.6 years, with a higher prevalence in females (83.1%). No significant difference was found between the affected knee sides. Grade 3 was the most frequent in both the US‐based severity assessment and KL grade distribution, occurring in 58.0% and 48.2% of cases, respectively (Table 2).

**Table 2:** Participants′ data.

**Data**	**Result**
Age, mean ± SD	61.40 ± 10.64
Gender, *n* (%)	
Male	19 (16.9%)
Female	93 (83.1%)
Knee affected side, *n* (%)	
Right	57 (51.8%)
Left	55 (49.1%)
US‐based knee OA severity assessment, *n* (%)^a^	
Grade 0	2 (1.7%)
Grade 1	12 (10.7%)
Grade 2	19 (16.9%)
Grade 3	65 (58.0%)
Grade 4	14 (12.5%)
Kellgren–Lawrence Grade, *n* (%)	
Grade 0	2 (1.7%)
Grade 1	14 (12.5%)
Grade 2	30 (26.7%)
Grade 3	54 (48.2%)
Grade 4	12 (10.7%)

^a^Weighted kappa of US‐based knee OA severity assessment between two raters showed *κ* = 1.00.

### 3.2. Descriptive Analysis

Descriptive analysis was conducted by calculating sensitivity, specificity, PPV, NPV, and accuracy for each grade, as presented in Table 3. These values were summarized to describe the diagnostic performance of US compared with CR across different grades, not as a statistical test.

**Table 3:** Descriptive analysis of US vs. CR to detect knee OA severity according to KL grading, including sensitivity, specificity, positive predictive value (PPV), negative predictive value (NPV), and accuracy.

**Grade**	**Sensitivity (%)**	**Specificity (%)**	**PPV (%)**	**NPV (%)**	**Accuracy (%)**
0	100.0	100.0	100.0	100.0	100.0
1	71.4	98.0	83.3	96.0	94.6
2	50.0	95.1	78.9	83.9	83.0
3	87.0	69.0	72.3	85.1	77.7
4	66.7	94.0	57.1	95.9	91.1

### 3.3. Interrater Analysis

Interrater reliability between the two US raters was assessed using a weighted kappa analysis. The analysis showed a weighted kappa value of 1.00. These results indicate near‐perfect agreement between the two raters, suggesting good reproducibility of US scans in this study.

### 3.4. Association Between US‐Based Knee OA Severity Assessment and KL Grading

The statistical analysis demonstrated a significant association between the US‐based knee OA severity assessment and KL grading, as determined by a chi‐square test with *p* value of < 0.001. In addition, Cramér′s *V* test was analyzed, and the result was 0.742 (95% CI: 0.599–0.824), which indicates a very strong association between US‐based knee OA and KL grading.

## 4. Discussion

This study demonstrated a significant association between the severity of knee OA, as assessed by the US‐based knee OA severity assessment, and the KL grading in 112 participants with knee OA. These findings indicate that knee OA US scanning is well associated with the KL grading. Mortada et al. compared the KL grading scale with US and reported that the US grading scale demonstrated higher overall sensitivity (94.6%) and specificity (93.3%) for identifying various grades of knee OA [13]. The results of this study align with previous research, which found a significant association between KL grading and various US grading systems [9]. Compared with CR, US is superior or at least equally effective in detecting soft tissue abnormalities, such as synovial hypertrophy, joint effusion, osteophytes, medial meniscus prominence, and changes in the medial FC [7, 19].

In this study, the primary parameters assessed were FC, with a focus on their regularity. Cartilage thickness loss frequently occurs in older adults, with or without knee OA, and serves as an important biomarker for knee OA development [20]. Cartilage degradation, a hallmark of OA, is considered a progressive process, though it remains unclear whether this process is reversible or irreversible [21]. Our previous study evaluating FC thickness in knee OA patients and a healthy population found that the OA group had significantly thinner FC, on average, compared with the healthy group [11]. Additionally, another study showed that cartilage thickness loss is significantly associated with a slight worsening of pain over 24 months. The association between cartilage thickness loss and pain is mediated by synovitis‐related changes [22].

This study also evaluated the presence of osteophytes and meniscus protrusion in the MKJ and LKJ as additional parameters. According to the KL grading system, osteophytes begin to appear at Grade 2 and become increasingly severe with higher grades. Similarly, the US‐based knee OA severity assessment in this study evaluates the presence of osteophytes beginning at Grade 2 or higher. These findings support previous studies comparing US with CR, concluding that US performs better in detecting osteophytes on the tibiofemoral side, FC degeneration, and medial meniscal extrusion. Furthermore, US is also more sensitive for detecting joint effusion and synovitis [23, 24]. A review article demonstrated that US is more sensitive than CR in detecting tibiofemoral osteophytes [25] CR may identify osteophyte formation, joint space narrowing, subchondral bone thickening, and cyst formation; however, these radiographic findings do not strongly correlate with knee pain [26]. A similar study involving 86 participants who underwent US scanning for knee OA found a significant correlation between the presence of osteophytes and meniscus extrusion and KL grading severity [27].

Compared with CR, US provides additional diagnostic value by directly visualizing structural abnormalities in the meniscus and cartilage and detecting effusion, synovitis, and tibiofemoral osteophytes with high sensitivity. The advantages of US over radiography include its ability to image soft tissue structures and detect small or early lesions [25]. US is an affordable and accessible imaging technique that facilitates the assessment of both structural and inflammatory changes in OA joints. Based on the reviewed literature, US can serve as an adjunctive diagnostic tool to CR in clinical OA diagnosis. Furthermore, ongoing technological advancements are expected to enhance the diagnostic utility of US in the future [7]. Even in studies evaluating cartilage at the microscopic level, US assessments of cartilage morphology have shown strong correlations with histologic grading [28]. The results of this study indicate strong agreement between US‐based grading and KL grading for Grades 1 and 2. As a clinical implication, this US‐based knee OA severity assessment proves to be a valuable tool for evaluating cartilage in knee OA patients.

However, this study has several limitations. First, despite achieving very high interrater reliability, US scanning remains operator‐dependent, which poses potential interpretation bias in assessing the severity of knee OA. Second, this study did not cover long axis scanning of medial and lateral FC. Therefore, we recommend using long‐axis US scanning for future studies. Third, another potential source of bias in this study is that the anatomical regions evaluated by the US and CR were not identical, which may affect diagnostic agreement.

## 5. Conclusion

US‐based knee OA severity assessment demonstrated a significant association with the KL grade. The near‐perfect interrater agreement suggests strong reproducibility of this method. The precision of US in assessing knee OA severity presents distinct advantages over CR.

## Ethics Statement

Ethical approval was granted by the Medical Research Ethics Committee of RSUD Haji Provinsi Jawa Timur, Surabaya, Indonesia, under Approval Number 445/106/KOM.ETIK/2024. The study commenced after obtaining informed consent from each participant. This study obtained verbal consent from all participants. No identifying information was recorded, and confidentiality was maintained throughout the study.

## Disclosure

All authors approved the final manuscript.

## Conflicts of Interest

The authors declare no conflicts of interest.

## Author Contributions

R.V.P.: conceptualization, methodology, data collection, data analysis, drafting, writing, and reviewing; H.: methodology, writing, and reviewing; A.: drafting, writing, and reviewing; N.H‐D.: data collection, data analysis, writing, reviewing; A.Z.Z.A.H.: data analysis, drafting, writing, reviewing, and other administration; A.N.R.: Conceptualization, writing, and reviewing.

## Funding

No funding was received for this manuscript.

## Data Availability

The data availability can be requested through the corresponding author contact.
